# Design and Implementation of 2.45 GHz Passive SAW Temperature Sensors with BPSK Coded RFID Configuration

**DOI:** 10.3390/s17081849

**Published:** 2017-08-10

**Authors:** Chen Fu, Yabing Ke, Min Li, Jingting Luo, Honglang Li, Guangxing Liang, Ping Fan

**Affiliations:** 1 Shenzhen Key Laboratory of Advanced Thin Films and Applications, College of Physics and Energy, Shenzhen University, Shenzhen 518060, China; chenfu@szu.edu.cn (C.F.); limin@szu.edu.cn (M.L.); lgx@szu.edu.cn (G.L.); fanping@szu.edu.cn (P.F.); 2 Institute of Acoustics, Chinese Academy of Sciences, Beijing 100190, China; keyabing@mail.ioa.ac.cn

**Keywords:** surface acoustic wave (SAW) device, passive, radio frequency identification (RFID), temperature sensor

## Abstract

A surface acoustic wave based passive temperature sensor capable of multiple access is investigated. Binary Phase Shift Keying (BPSK) codes of eight chips were implemented using a reflective delay line scheme on a Y-Z LiNbO_3_ piezoelectric substrate. An accurate simulation based on the combined finite- and boundary element method (FEM/BEM) was performed in order to determine the optimum design parameters. The scaling factor ‘s’ and time delay factor ‘τ’ were extracted using signal processing techniques based on the wavelet transform of the correlation function, and then evaluated at various ambient temperatures. The scaling factor ‘s’ gave a more stable and reliable response to temperature than the time delay factor ‘τ’. Preliminary results show that the sensor response is fast and consistent subject to ambient temperature and it exhibits good linearity of 0.9992 with temperature varying from 0 to 130 °C.

## 1. Introduction

Passive and wireless sensing has attracted considerable attention in the field of detection and monitoring in harsh or inaccessible environments, such as with explosive, corrosive, high vacuum, extreme temperature and high radiation level characteristics. Passive sensors based on surface acoustic wave (SAW) technology have been reported as a favorable solution due to their low cost, small size and reproducibility [1,2]. SAW-based wireless and passive sensors have been widely used for temperature [3,4,5], pressure [6], strain [7,8], torque [9], magnetic field [10], and corrosion [11] sensing applications. In particular, SAW temperature sensors have been regarded as one of the most popular applications, which are able to physically measure temperatures up to 1000 °C remotely and need no power source [3,12,13]. In addition, they can withstand shock levels exceeding 1000 g and are inherently immune to ionizing radiation [14]. There are two types of SAW-based sensor structures: resonant [15,16] and delay line types [17,18].

**Figure 1:**
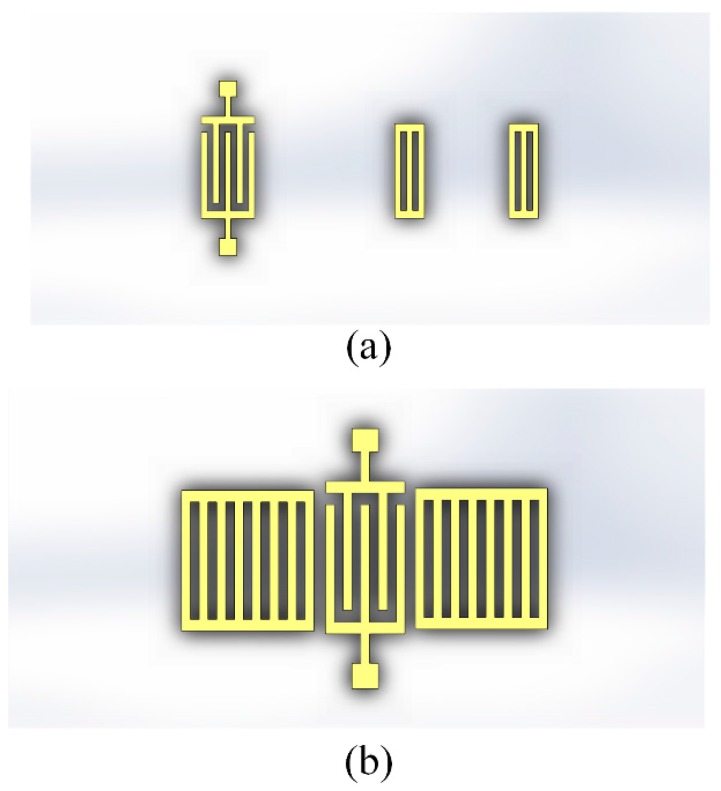
Schematic diagrams of (**a**) Reflective delay line and (**b**) Resonator surface acoustic wave (SAW) sensors.

Meanwhile, wireless sensor network technology has been engaging the effort of many researchers in the past two decades [19]. It demands a large number of low power and cheap sensor nodes to build up smart intelligent grids to simultaneously monitor different parts of a desired area [20,21]. With regards to wireless sensor networks, it is essential to differentiate one particular sensor of interest from any others that might be within the range of the interrogator. Radio frequency identification (RFID) tag technology enables the unambiguous recognition of every node and simultaneous readings of many nearby ones in a vast sensor network, followed by deconvolution of the resulting signal. SAW-based RFID tags have been proposed for mass application due to their advantages of low cost, small size, low radiation power and robustness [18]. In particular, SAW tags, which can easily incorporate sensor functions, show an inherent capability for operation as sensors [22]. To date, SAW RFID built-in temperature sensors have been widely investigated with a variety of structures [23,24,25] and materials [26,27]. To date, SAW wireless and passive sensors are designed as two types of configuration as reflective delay lines and resonators, for which schematic diagrams are respectively depicted in Figure 1. The reflective delay line utilizes several reflectors located at different distances from the interdigital transducers (IDTs). The SAW covers the distance between the IDT and reflectors twice and the differential amplitude/time in time domain is measured. Some SAW sensors working at 433 MHz frequency have been reported using reflective delay line configurations [28,29,30]. However, these sensors required a wide frequency band up to 100 MHz, which massively exceeds the ISM standard. Instead, it is preferable for most 434MHz SAW wireless sensors to use the resonator scheme [31,32]. This utilized two differential resonators as sensor elements which share sensitivity to other ambient conditions but have different sensitivity levels to the desired measurement condition.

Recently, the demand for miniature sensors with high sensitivity has encouraged the development of the technology towards higher frequency, normally in the ISM range of 2.45 GHz. Reflective delay line configurations have more advantage due to making use of the corresponding 80 MHz bandwidth and thus these have attracted more and more attention. Aiming at the optimal design of the reflective delay line, high efficiency modulation schemes play a key role in improving its encoding capacity and sensitivity. In general, orthogonal frequency code (OFC) [33] and pulse position [34] and phase modulations [35] have been reported as three typical encryption methods to encode SAW-based reflective delay lines. The OFC code can provide low loss and offer greater range, but suffers from relatively large size and complex reflectors [36]. Pulse position coded sensors show much lower sensitivity than phase coded ones. However, the issue of phase ambiguity has prevented the phase evaluation of sensors operating over a range larger than 2π [37].

**Figure 2:**
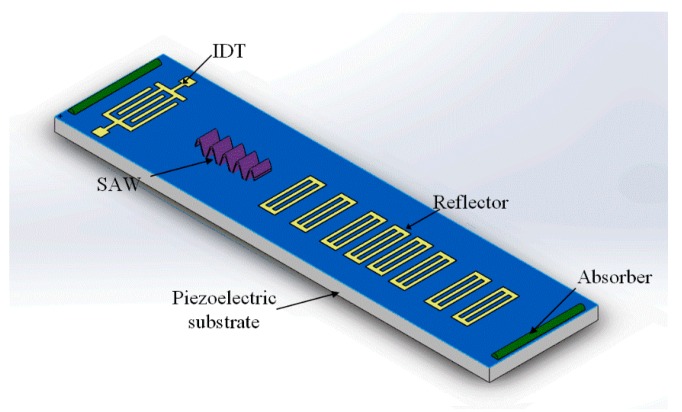
Schematic diagram of the wireless and passive SAW temperature sensor.

In this paper, the objective is to design an ID-tag type coded wireless and passive SAW temperature sensor operating at the 2.45 GHz band. The schematic is shown in Figure 2. A single carrier RF burst is emitted to the IDT and generates an SAW that propagates toward the reflectors, and is then partially reflected back. The reflectors are arranged according to binary phase shift keying (BPSK) code and the resulting reflected pulses indicate the device code and the measured temperature. Optimal structure simulation for the reflective delay line is carried out prior to fabrication. We introduce a practical algorithm to extract the scaling factor ‘*s*’ and delay factor ‘τ’ from the wavelet transform of the received signal, which are used to evaluate the temperature. The fundamentals, methodology, implementation and experimental results of the SAW sensors are demonstrated in detail.

## 2. Design Considerations and Optimization

**Figure 3:**
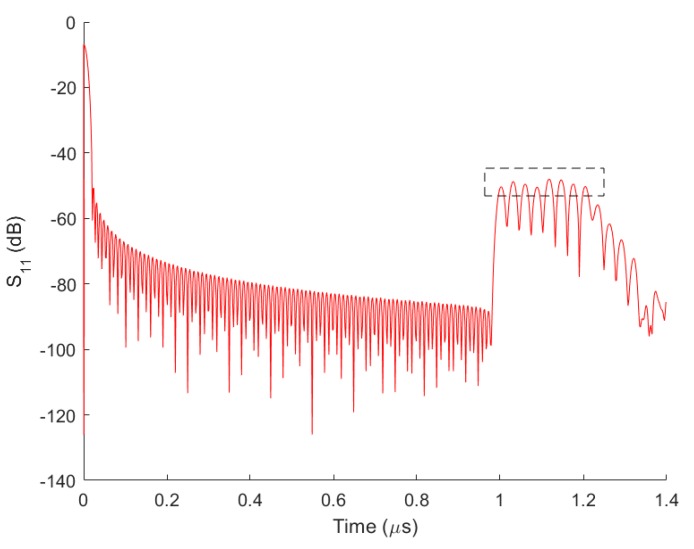
Simulated S11 amplitude response of the SAW sensor with the reflector design parameters in Table 1.

Y-Z LiNbO_3_ was used as the substrate material because of its large electromechanical coupling and high temperature coefficients. An initial 2 mm chip space was set between the IDT and the first reflector to isolate environmental echoes. The duration of a reflector pulse was equal to two times the length of the IDT plus that of the reflector. To avoid overlap among neighboring impulses, the minimum gap of the reflectors is set to be

(1) $$\Delta L=\frac{T_{s}V}{2}=L_{\mathrm{IDT}}$$

where the Ts is the time position difference of the neighboring reflectors, V is the velocity of the SAWs and LIDT is the length of the IDT. The metallization ratio is designed to be a fixed value of 0.5. The aluminum thickness is set at 50 nm as a trade-off between large reflection coefficient and bulk wave suppression. In order to minimize reflectivity loss, the 8 chip BPSK-coded scheme was adopted and all reflectors were designed with open-circuit electrodes. The 180° phase was realized by shifting 1/4 wavelength of the reflector location. Aiming for the amplitudes of all echoes to be uniform, the finger number of reflectors is progressively adjusted. The IDT consisted of 21 finger pairs. The wavelength and aperture of IDT and reflectors were uniformly 1.352 and 82.50 µm. Table 1 depicts the reflector design parameters of the SAW sensor. Moreover, FEM/BEM [38,39] was used to simulate the corresponding response of the sensor, which is shown in Figure 3. Eight reflection peaks appear in the time window ranging from 1.11 to 1.21 µs, which stand for the reflections of SAW from those eight reflector gratings. In addition, several relatively weaker peaks emerging on the right are due to multi-reflections among those reflectors.

## 3. Correlation Processing Algorithm

The wavelet transform (WT) of the received signal with respect to differently scaled versions of the mother wavelet is determined as a function of a scaling factor ‘***s***’ and time delay factor ‘*τ*’, as formulated below [40],

(2) $$W_{fg}\left(s,\tau \right)=\frac{1}{\sqrt{\left|s\right|}}\overset{\infty }{\underset{-\infty }{\int }}g\left(t\right)f^{*}\left(\frac{t-\tau }{s}\right)dt$$

where *W_fg_* is the designated ambiguity function and the functions g and f are the received signal and mother function respectively. The wavelet transform represents a signal in time domain in terms of time delay and the scaled domains of a mother function signal. The response at a fixed temperature of 25 °C is taken as the mother function. For each interrogation, the receiver needs to calculate the correlation and ambiguity function with varying scaling factor *‘s’* and delay time *‘τ’*. The maximum peak of the ambiguity function is selected and the corresponding values of s_m_ and τ_m_ denote the matching scaling factor and time delay.

**Figure 4:**
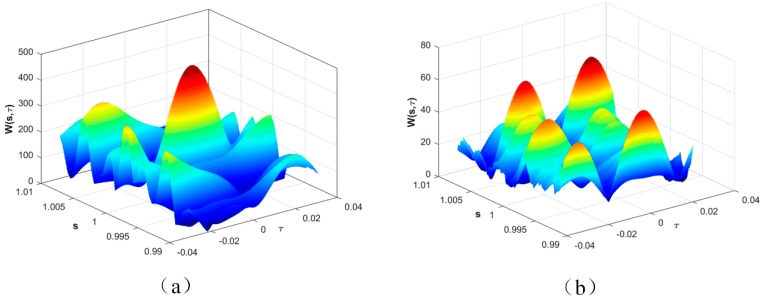
3D plot of ambiguity functions of the temperature sensor response at 25 °C (**a**) and 100 °C (**b**) respectively.

Since temperature variation causes a mechanical elongation or compression and changes in the material constants of the substrate and also results in a change in its SAW velocity, the SAW impulse-response waveforms are shifted and scaled in time at the receiver. In operation, all the responses of S_11_ in the time domain were firstly truncated to only preserve the range from the first reflector’s impulses to the last. Then the truncated signal at the temperature T_0_ (room temperature, 25 °C) was chosen as the designated mother function. The S_11_ at an arbitrary temperature was taken as the received signal function. The corresponding ambiguity function was calculated and matching scaling factor and the time delay were determined in the presence of additive white Gaussian noise (AWGN) at that temperature. Figure 4a shows the ambiguity function at room temperature in terms of time delay and scaling factors respectively. It is observed that the maximum peak appears at values of *s* = 1 and *τ* = 0. This agrees with the self-correlation or self-matching prediction as its response was taken as the designated mother function. In contrast, the maximum peak shifts to the locations of *s* = 1.006 and *τ* = 0.24, as is shown in Figure 4b. Similarly, the particular ambiguity functions of various temperatures can be calculated and their values of s_m_ and τ_m_ extracted accordingly. Two methods were used to evaluate the measured temperature based on either the matching scaling factor or time delay.

## 4. Fabrication and Implements

**Figure 5:**
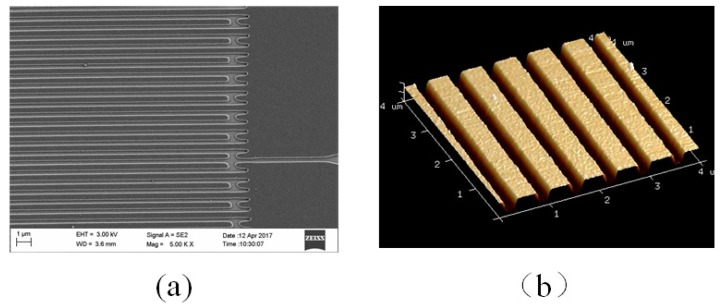
SEM (**a**) and AFM (**b**) characterisations of a section of the fabricated interdigital transducer (IDT).

Prototype SAW sensor devices for this project were fabricated on 4”, 500 µm thick single-polished Y-Z cut LiNbO_3_ wafer. The SAW IDTs consist of aluminum electrodes via E-beam lithographical printing on the Y-Z LiNbO_3_ substrate. AFM (atomic force microscope) and SEM (scanning electron microscopy) were used to inspect the IDT and reflector patterns. Figure 5 presents an SEM micrograph of a segment of the fabricated IDT pattern. Those finger patterns were successfully made and this proves that the E-beam technique is adequate for the device with a minimum feature size of about 400 nm. The height of aluminum fingers was measured at about 45 nm. The SAW device was affixed in a surface mount device (SMD) package and electrically bonded for testing.

**Figure 6:**
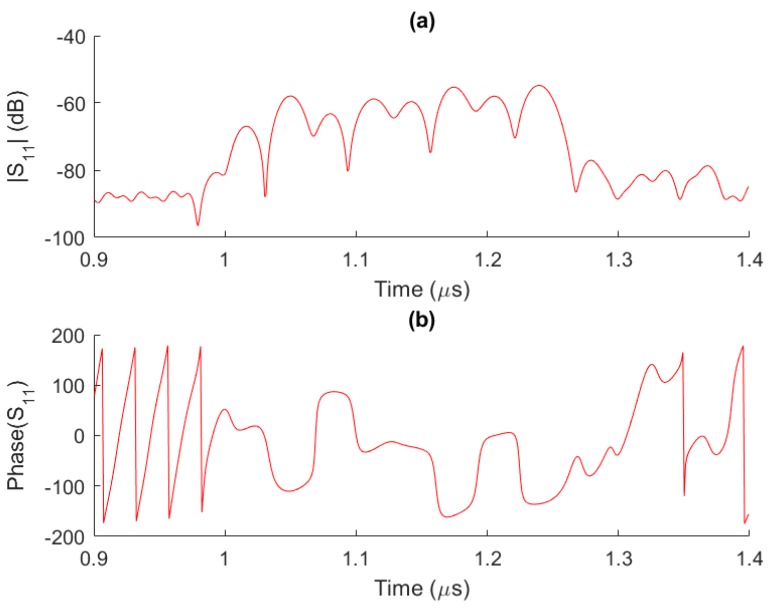
Amplitude (**a**) and phase (**b**) responses of the fabricated sensor in the time domain.

The sample was then sealed in the SMD package to avoid the ambient contamination. The reflection loss (S_11_) of the fabricated chip was measured with a vector network analyzer and then its response lower than 0.9 or greater than 1.4 µs was truncated. The corresponding amplitude and phase responses in the time domain are shown in Figure 6. Approximately eight uniform peaks are observed, which is in line with the designed eight reflectors. The tiny distortions present are attributed to multi-reflections among these reflectors.

**Figure 7:**
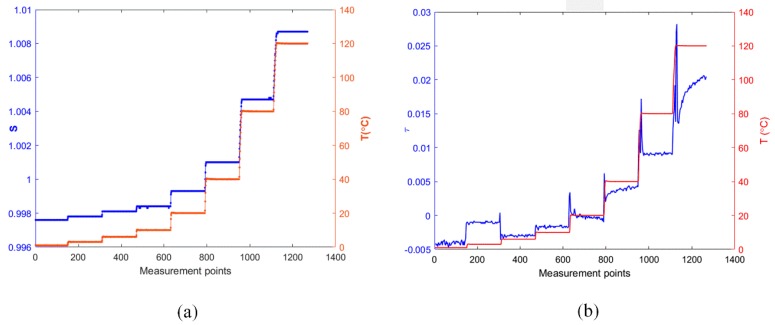
Real time responses of S scaling (**a**) and τ (**b**) parameter.

Then the sensor was tested in an oven with a temperature control accuracy of 0.1 °C. The temperature in the oven was set to eight values in the range of 0 °C to 120 °C and each temperature was held for a duration of 3 minutes. The response data was collected with one second sample intervals. Meanwhile, both *s* and *τ* parameters were calculated at each point accordingly based on the aforementioned correlation algorithm, and the results are shown in Figure 6. It is observed that *s* and τ both perform the expected trend subject to the change in ambient temperature. As is shown in Figure 7b, the response of τ presents more obvious disturbance, particularly at the temperature transition points. In addition, its linearity also appears much lower. In contrast, as the temperature stabilizes, the measured *s* response tends to stabilize and shows promising stability at the various temperatures. Also, it shows consistent shifts to the temperature transitions, as is shown in Figure 7a. Therefore, the parameter *s* has advantages over τ in terms of temperature evaluation.

**Figure 8:**
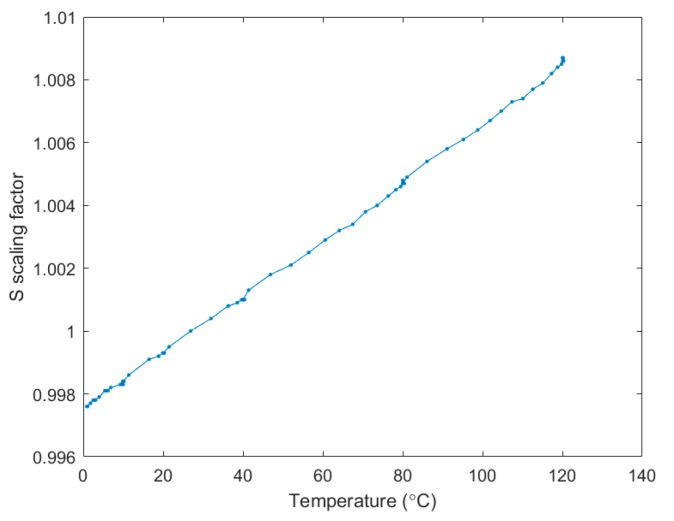
Measured sensor response versus temperature from 0 °C to 120 °C.

Figure 8 depicts the S scaling parameter response as a function of the temperature variation. The sensor response is largely proportional to the ambient temperature inside the oven. The slope of the fitting data, which is the well-known linear temperature coefficient of delay (TCD), can be calculated as:

(3) $$\mathrm{TCD}=\;\frac{1}{S}\frac{\partial S}{\partial T}$$

The TCD of *s* factor was characterised as 92.15 ppm/°C, which is close agreement with the values from the literature [13,41]. The root mean square (RMS) of the *s* term fitting is equal to 1.027 × 10^−4^, which corresponds to an RMS of temperature of 1.110 °C. Since the oven volume was much bigger than the sensor size, the display value of the oven was the average temperature inside rather than the precise value at exact location of the sensor. Besides this, the measured fluctuation is also attributed to the low level of air flow inside the oven. Despite the limitations and the unfavourable issue associated with the test oven, the good linearity and sensitivity found still prove that the scaling factor evaluation method is reliable and feasible for temperature monitoring. It also shows detection limit to very tiny temperature fluctuation in the oven as well as excellent stability.

**Figure 9:**
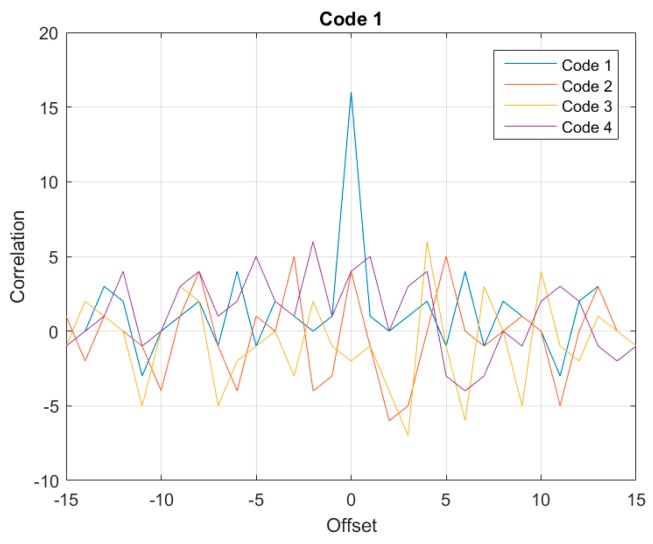
Correlation of four 16-bit Gold codes.

For the further application of multi-sensor network, Gold codes are used to distribute to the sensor nodes. For a code with 16 bits, the correlation functions are shown in Figure 9. The autocorrelation peak value should be 16. Side lobe level of 6 indicates those codes that interaction induces roughly 1/3 of the autocorrelation peak. This scheme with low cross-correlation can be used to implement sensor diversity in passive and wireless SAW multi- sensor systems, operation in systems with correlation-based transceivers.

## 5. Conclusions

Reflective delay lines capable of wireless interrogation in the ISM band from 2.4 to 2.48 GHz were designed and fabricated on Y-Z cut LiNbO_3_ wafer. The correlation function algorithm was demonstrated to evaluate the tested temperature. The method to utilize the scaling factor *s* was proven more stable and reliable than utilizing the factor *τ*. The experiments showed that the sensor behaved with good linearity and low detect limit. The results achieved in the research illustrate that the detection scheme works perfectly with wireless and passive SAW temperature sensors. Although this work is currently configured only to measure temperature, it will next be applied SAW sensors for other parameter with appropriate revision, such as for pressure, toxic gases and torque.

## Figures and Tables

**Table 1:** Reflector design parameters of the reflective delay line sensor.

	Position (µm)	Number of Fingers
1st	1755	4
2nd	1805	5
3rd	1855	5
4th	1905	5
5th	1955	7
6th	2005	8
7th	2055	9
8th	2105	11
